# Upregulation of *miR-17-92* cluster is associated with progression and lymph node metastasis in oesophageal adenocarcinoma

**DOI:** 10.1038/s41598-019-48624-0

**Published:** 2019-08-20

**Authors:** Patrick Sven Plum, Ute Warnecke-Eberz, Uta Drebber, Seung-Hun Chon, Hakan Alakus, Arnulf Heinrich Hölscher, Alexander Quaas, Christiane Josephine Bruns, Ines Gockel, Dietmar Lorenz, Ralf Metzger, Elfriede Bollschweiler

**Affiliations:** 1 Department of General, Visceral and Cancer Surgery, University of Cologne, Faculty of Medicine and University Hospital Cologne, Kerpener Straße 62, D-50937 Cologne, Germany; 2 Institute of Pathology, University of Cologne, Faculty of Medicine and University Hospital Cologne, Kerpener Straße 62, D-50937 Cologne, Germany; 3 Center for Oesophageal and Gastric Surgery, AGAPLESION Markus Krankenhaus, Wilhelm-Epstein-Straße 4, D-60431 Frankfurt am Main, Germany; 4 Department of Visceral, Transplant, Thoracic and Vascular Surgery, University Medical Center Leipzig, Liebigstraße 20, D- 04103 Leipzig, Germany; 5 Department of General, Visceral and Thoracic Surgery, Klinikum Darmstadt GmbH, Grafenstraße 9, D-64283 Darmstadt, Germany; 6 Department of General, Visceral, Thoracic and Cancer Surgery, CaritasKlinikum Saarbrücken, Rheinstraße 2, D-66113 Saarbrücken, Germany

**Keywords:** Prognostic markers, Surgical oncology

## Abstract

The occurrence of lymph node metastasis (LNM) and depth of tumour infiltration are significant prognostic factors in oesophageal adenocarcinoma (OAC), however no reliable prognostic biomarkers have been established so far. Aim of this study was to characterize microRNAs (miRs) of OAC patients, who primarily underwent oesophagectomy, in order to identify specific alterations during tumour progression and LNM. MicroRNA array-based quantification analysis of 754 miRs, including tumour specimens of 12 patients with pT2 OAC from three different centres (detection group), was performed. We identified *miR-17*, *miR-19a/b*, *miR-20a*, and *miR-106a*, showing the best predictive power for LNM. These miRs were validated by quantitative real time-PCR (qRT-PCR) in 43 patients with different tumour stages (pT1: n = 21; pT2: n = 12 and pT3: n = 10) (training group) (*p* < 0.05), demonstrating that increasing levels of identified miRs were associated with advanced depth of tumour infiltration. These findings were verified in another independent group of 46 pT2 OAC patients (validation group). Quantitative RT-PCR analysis of the miR-panel confirmed these results except for *miR-19a* (*p* < 0.05 each). Logistic regression analysis identified *miR-17* and *miR-20a* (*p* = 0.025 and *p = *0.022, respectively) to be independent variables for prediction of LNM. The mathematical prediction model was used in the validation group, and the estimated prognosis was compared to the actual postsurgical follow-up. This comprehensive data demonstrated the importance of *miR-17-92* cluster and *miR-106a* for progression as well as LNM in OAC indicating that those might be feasible prognostic biomarkers.

## Introduction

Oesophageal adenocarcinoma (OAC) is a severe neoplasia with an overall 5-year survival rate ranging from 15% to 25%^1,2^. Within western countries, its incidence is still rising^3–6^. The occurrence of lymph node metastasis (LNM) is one of the major risk factors resulting in a poor prognosis. Pre-interventional diagnostic methods such as computer tomography (CT) or endosonographic ultrasound (EUS) are used to examine the absence of LNM. Despite the high accuracy in tumour invasion depth, the prediction of LNM is extremely poor since nodal micro-metastasis or extra-nodal lymph node metastasis are phenomena that cannot be detected by these procedures^7^. Since recent analysis demonstrated that LNM occurs even in early stages of OAC limited to the mucosa (0–8.1% risk) (pT1a) or submucosa (20–30% risk) (pT1b), pretherapeutic prediction of lymph node involvement is mandatory^8–12^. Nevertheless, there is a lack of pretherapeutic biomarker-based evaluation indicating an aggressive tumour biology to better identify patients at risk before loco-regional or disseminated metastasis becomes clinically evident.

MicroRNAs (miRs) are non-coding, 21–25 nucleotide small RNAs that regulate gene expression by inhibiting translation^13^. After transcription and splicing within the nucleus and their transfer into cytoplasm, pre-miRs are processed into the mature miRs. Depending on the level of sequence complementarity, the miRs act on specifically targeted messenger RNAs (mRNAs) either by translational repression or mRNA cleavage^13,14^. More than 1500 miRs have been described as being involved in the tumorigenesis of several malignancies by upregulated oncogenetic or decreased oncosuppressive effects on proliferation, tumour cell survival, migration, metastasis, or angiogenesis^14–18^. In addition, there is evidence that miRs are feasible predictors for the prognosis of oesophageal cancer^19–21^.

*MiR-17*, *miR-19a/b*, and *miR-20a* are members of the *miR-17-92* cluster, which consists of six mature miRs (*miR-17*, *miR-18a*, *miR-19a*, *miR-20a*, *miR-19b-1*, and *miR-92-1*), also known as *oncomiR-1*. This cluster shows two paralogs within the human genome: *miR-106a* and *miR-106b*^22^. Both are overexpressed in several malignant cancers (including oesophageal cancer) and are suspected to be associated with oesophageal cancer radioresistance^23,24^.

Since miRs are promising biomarker candidates for prediction and therapeutic response in different neoplasms, this study aimed to characterize miR profiles of OAC patients, not treated with neoadjuvant therapy, to identify putative alterations associated with the progression or occurrence of LNM. The identified miRs were validated regarding to their predictive impact on OAC.

## Results

### Patient characteristics

A total of 89 patients who underwent primary oesophagectomy due to OAC were included in this study and divided into three groups: detection, training and validation group. There were 12 pT2 patients with either pN0 (n = 6) or pN+ (n = 6) within the detection cohort while the training cohort included 43 patients with pT1-3 and both, pN0 and pN+ (including those patients of the detection cohort). In the independent validation cohort, only 46 pT2 patients with or without LNM were considered. Our data was comparable to those of all patients who underwent an oesophagectomy at the other three centres and histopathological baseline characteristics of all patients included within the current analysis are summarized within Table 1.

**Table 1:** Histopathological baseline characteristics of all patients included.

Factor	Detection group	Training group	Validation group
total (n)	12	43 (including detection group)	46
**Age**			
median	61 years	63 years	66 years
(min-max)	(48–77 years)	(38–79 years)	(46–80 years)
**Gender**			
Male	11	39	38
Female	1	4	8
**T-category**			
pT1	0	21	0
pT2	12	12	46
pT3	0	10	0
**N-category**			
pN0	6	18	15
pN+	6	25	31

### Altered miR profiles depending on the lymphatic invasion of OAC

**Figure 1:**
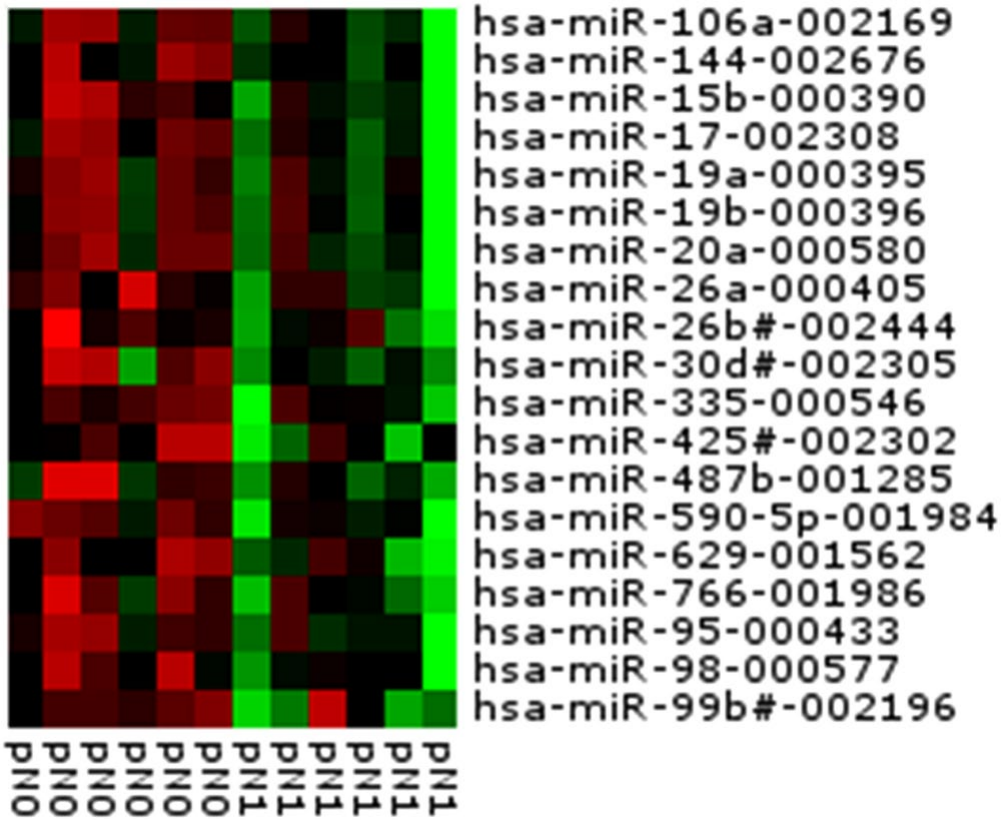
MicroRNA Array-based miR profiling within the detection cohort (n = 12). MiRs from 12 formalin-fixed paraffin-embedded (FFPE) samples derived from surgical pT2 oesophageal adenocarcinoma (OAC) specimens underwent miR-profiling of 754 miRs via MicroRNA Array. Intratumoural miR expression of the 19 most differently expressed miRs of OAC patients with and without lymph node metastasis (LNM) including five members of the miR-17-92 cluster (*miR-17*, *miR-19a/b*, *miR-20a*, and *miR-106a*) was visualized as a heat map.

To identify LNM-indicating candidate miRs, we profiled miR-expression of the 12 pT2 pN0/+ OAC patients (detection cohort) by using PCR-based miR analysis consisting of 754 different miRs. If miRs were detected in at least two independent tumour samples, they were considered for further analysis. 19 miRs showed the most differences in expression between patients with and without LNM (see heatmap in Fig. 1).

### *MiR-17-92* cluster and *miR-106a* and their correlation with lymphatic invasion

Among the eight most significantly different markers, five miRs were members of the *miR-17-92* cluster or its paralogue, *miR-106a*. Therefore, we focused on *miR-17*, *miR-19a/b*, *miR-20a*, and *miR-106a* for further analysis.

**Figure 2:**
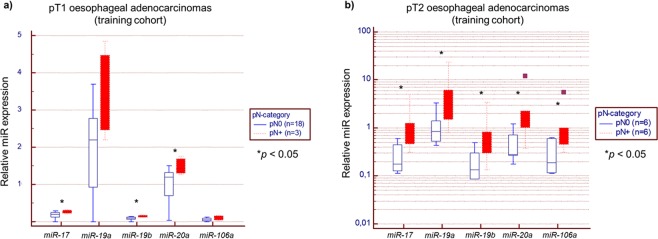
Intratumoural miR expression of *miR-17*, *miR-19a/b*, *miR-20a* and *miR-106a* in correlation to lymph node status within the training cohort (n = 43). Further examination of *miR-17*, *miR-19a/b*, *miR-20a*, and *miR-106a* was performed within an enlarged training cohort of 43 pT1-3 patients (pN0 versus pN+) who underwent primary oesophagectomy. Quantitative real time-PCR (qRT-PCR) analysis of the intratumoural miR expression (**a**) for patients with pT1 OAC and (**b**) for those with pT2 OAC tumours were compared to the corresponding pN status. *MiR-17*, *miR-19a/b* and *miR-20a* were significantly upregulated in pT1pN+ tumours (each p < 0.05) while all five miRs showed significant increase in pT2pN+ patients (each p < 0.05).

Single quantitative real time-PCR (qRT-PCR) examinations were performed to specifically validate those five miRs within the training cohort. Both, pT1 and pT2 OAC patients, showed a significant increase of miR levels in occurrence of LNM (*p* < 0.05 each, see Fig. 2a,b). However, there was only one patient with pN0 among ten pT3 patients so that reliable correlation between miR expression and status of nodal metastasis was not possible considering this tumour stage (data not shown).

**Figure 3:**
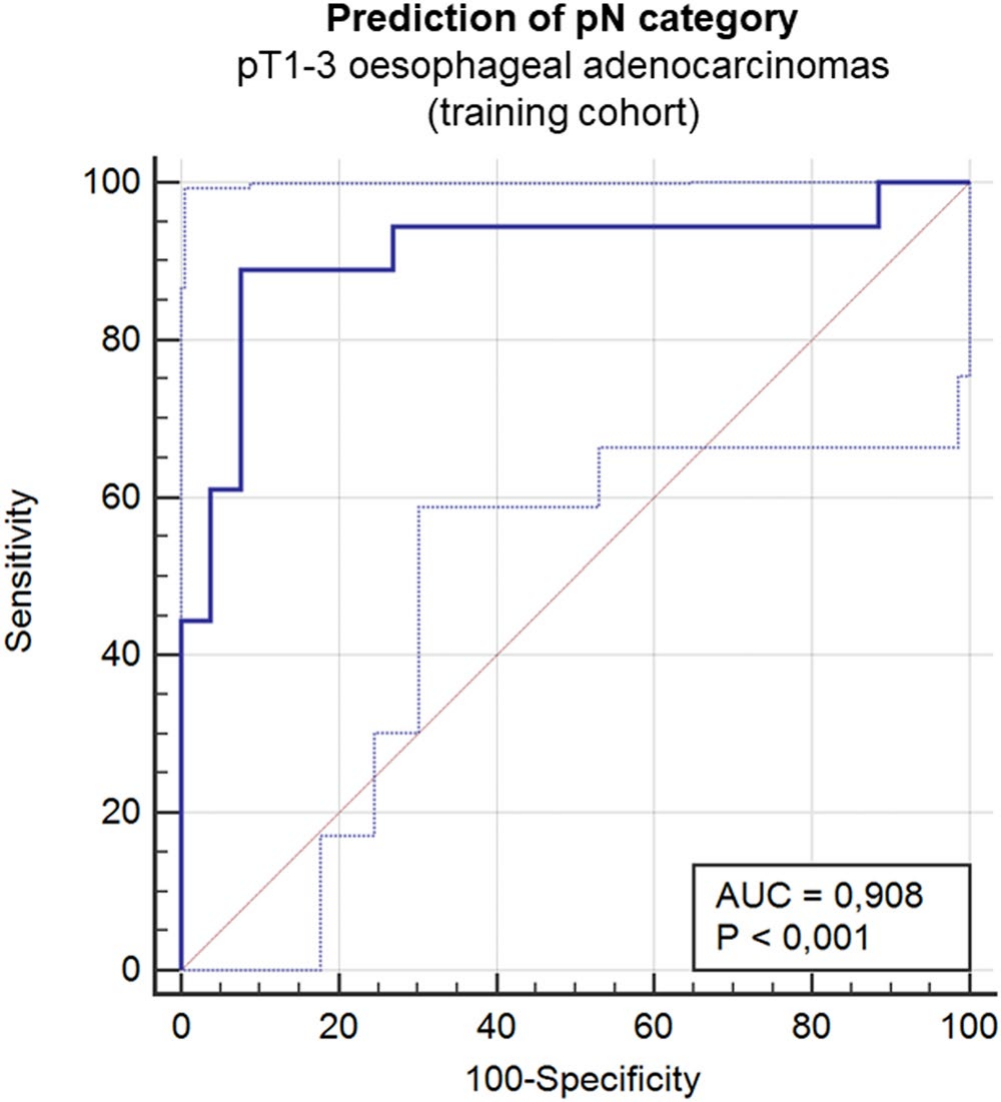
Design of a prediction model for lymph node metastasis (LNM) in the training cohort based on miR-17 and miR-20a. A prediction model was designed from logistic regression analysis with elimination of non-significant parameters in the training cohort (n = 43) utilizing *miR-17* and *miR-20a* as independent predictors for pN+ (*p* = 0.025 and *p* = 0.022). ROC analysis showed AUC = 0.908 (95% confidence interval: 0.782–0.974). Sensitivity was 88.89% and specificity 92.31% for a cut-off value of 0.632. Limits of the confidence intervals were illustrated as dotted lines within the graphic illustration.

For 43 patients with pT1-pT3 OAC of the training cohort, logistic regression analysis was performed including the five selected miRs to optimize the prediction of LNM. This statistical calculation model with backward elimination of non-significant markers showed a highly significant overall model fit (*p* < 0.0001) with logit (p) = −0.367 + 4.148 * miR17a −0.397 * miR20a. Independent variables for prediction of LNM were *miR-17* and *miR-20a* (*p* = 0.025, respectively *p* = 0.022). The “area under the curve” (AUC) within the receiver operating characteristic (ROC) analysis was AUC = 0.908 (95% confidence interval: 0.782–0.974) demonstrating a very close correlation between these two miRs and nodal metastasis within the cohort (Fig. 3). The sensitivity of this model was 88.89% and the specificity 92.31% for a cut-off value of 0.632.

### *MiR-17-92* cluster and *miR-106a* and their correlation with depth of tumour infiltration

**Figure 4:**
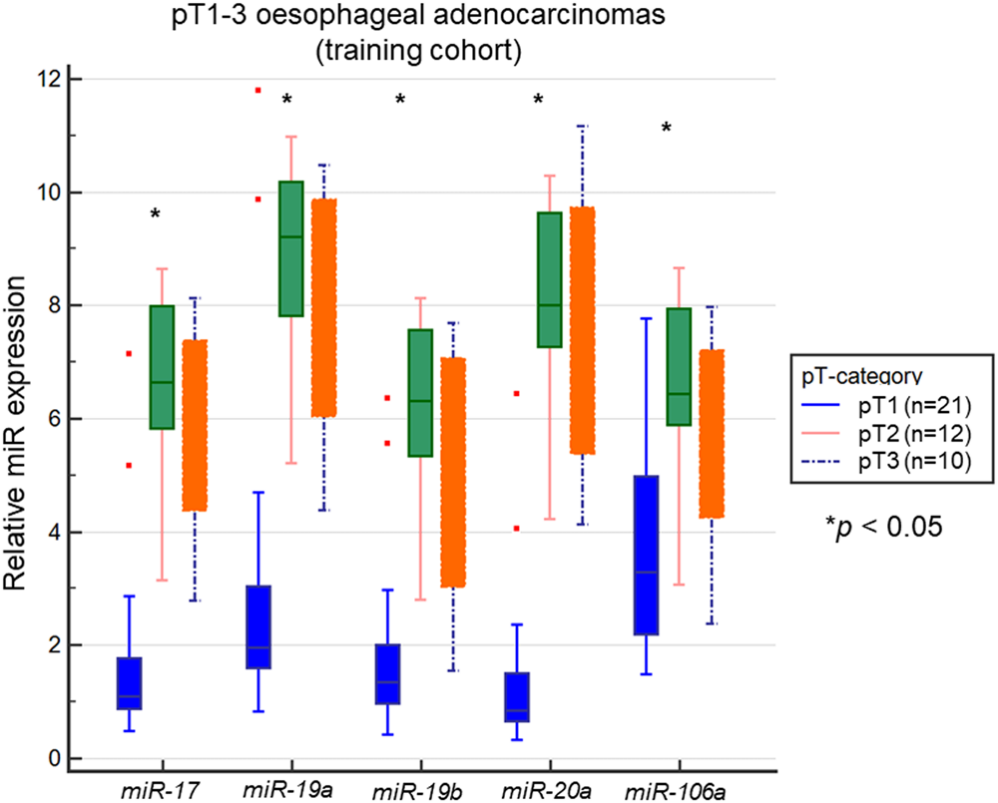
Intratumoural miR expression of *miR-17*, *miR-19a/b*, *miR-20a* and *miR-106a* in correlation to pT status within the training cohort (n = 43). Relative miR expression of *miR-17*, *miR-19a/b*, *miR-20a* and *miR-106a* was analysed in correlation to the pT-status within the training cohort (including pT1-3 tumours) using qRT-PCR and normalized to *snoU6*. M*iR-17*, *miR-19a/b*, *miR-20a*, and *miR-106a* were significantly decreased in pT1 OAC compared to locally advanced tumours (pT2-3) (each p < 0.05).

Analysing the results of the qRT-PCR of the training cohort revealed, that the panel of *miR-17*, *miR19a/b*, *miR-20a*, and *miR-106*a was significantly lower within early OAC limited to the mucosa or submucosa (pT1) compared to locally advanced stages such as pT2 or pT3 (see Fig. 4) (*p* < 0.05). Therefore, an upregulation of those miRs was associated with proceeding depth of tumour infiltration.

### Validation of the results in an independent group of patients with pT2 OAC

**Figure 5:**
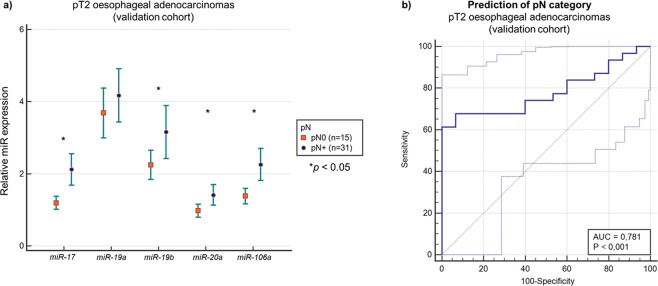
Intratumoural miR expression of *miR-17*, *miR-19a/b*, *miR-20a* and *miR-106a* in correlation to pN status within the validation cohort (n = 43). (**a**) Correlation between relative miR expression of *miR-17*, *miR-19a/b*, *miR-20a* and *miR-106a* and pN status of the independent 46 pT2 patients of the validation cohort confirmed upregulation of all miRs except for *miR-19a* in pN+ tumours using qRT-PCR (each p < 0.05). (**b**) Using the established prediction model for LNM on the validation cohort (n = 46) resulted in an AUC = 0.781 (95% confidence interval: 0.634–0.889) within the ROC analysis (p = 0.001). Sensitivity was 61.3% and specificity 100.0% with an optimal cut-off value of 0.725. Using the cut-off value of the training group (0.632), sensitivity was 67.7% and specificity 83.4%. Limits of the confidence intervals were illustrated as dotted lines within the graphic illustration.

An independent cohort of 46 consecutive patients with pT2 OAC (validation cohort) was used for further validation of the *miR-17-92* cluster. Quantitative RT-PCR analysis of these specific miRs confirmed the results for all miRs of this panel except *miR-19a* (*p* < 0.05 each) demonstrating elevated intratumoural miR expression in patients with LNM (see Fig. 5a).

In the second step, the above-mentioned prediction model of the training cohort for pN+ was applied to the validation cohort by calculating the predictive value for pN+ using the same equation as in the training group. Here, the AUC was 0.781 (95% confidence interval: 0.634–0.889) within the ROC analysis, p = 0.001 (see Fig. 5b). The sensitivity of this model was 61.3% and the specificity 100.0% with an optimal cut-off value of 0.725. The sensitivity of the model was 67.7% and the specificity 83.4% for the cut-off value of the training group of 0.632.

### Prognostic relevance of the *miR-17-92* cluster

**Figure 6:**
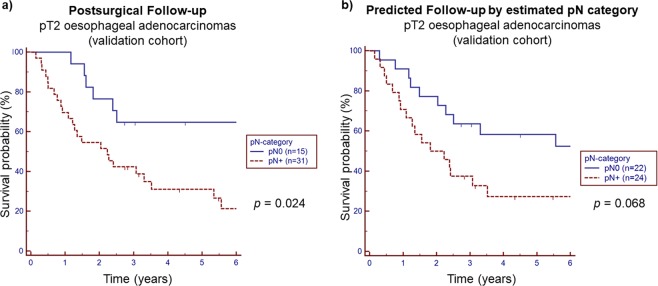
Prognostic impact of the *miR-17* and *miR-20a* for lymph node metastasis (LNM) within the validation cohort (n = 46). (**a**) Kaplan-Meier analysis demonstrated that LNM was significantly associated with poor postsurgical outcome in those pT2 patients of the validation cohort (p = 0.024). (**b**) Applying the prediction model for LNM based on *miR-17* and *miR-20a* led to a pN+ status in 24 patients versus 22 patients with pN0 within the validation cohort. Resulting Kaplan-Meier analysis of predicted postsurgical prognosis showed worse, but nonsignificant changes in survival rates for patients with estimated LNM (p = 0.068).

LNM was significantly associated with poor prognosis in the validation cohort resulting in a 5-year survival rate of 35% in pN+ OAC compared to 65% in pN0 patients within this group (*p* = 0.024) (see Fig. 6a).

Applying our mathematical prediction model to the validation cohort for calculating the predictive value of pN+ subdivided the group into 22 patients without suspected LNM and another 24 patients with suspected LNM. The corresponding 5-year survival rates were estimated to be 35% for pN+ and 60% for pN0 patients. Although the survival curves using this mathematical prediction were not significantly different (*p* = 0.068), the results were comparable to those derived from the histopathologically verified LNM of the patients’ actual postsurgical follow-up (see Fig. 6b).

## Discussion

The current study assesses the putative miR expression patterns in patients who underwent radical primary oesophagectomy due to OAC. We were able to demonstrate that the differently regulated expression of members of the *miR-17-92* cluster (*miR-17*, *miR-19a/b* and *miR-20a*) as well as its paralogue, *miR-106a*, were associated with either depth of tumour infiltration or presence of LNM. Particularly, *miR-17* and *miR-20a* were independent predictors of LNM in a mixed (pN0 and pN+) cohort of patients with pT1-pT3 OAC. Additionally, these results were confirmed in a further cohort of patients with pT2 OAC. Therefore, our findings support the role of these particular miRs as possible biomarkers for OAC. The influence of the *miR-17-92* cluster has been reported in several solid and hematogenous malignancies interfering in apoptosis, angiogenesis, tumour metabolism or proliferation^22,25–29^. However, only a small amount of data exists regarding the effects of the *miR-17-92* cluster in oesophageal cancer and this data mostly focuses on Asian cohorts and oesophageal squamous cell carcinoma (OSCC).

For therapeutic decision, knowledge about lymphatic infiltration is of high interest as the therapy is directly affected by a precise staging. In early local invasive cancers, an endoscopic resection can be applied in assumption of a very low risk of LNM (including early tumour stages up to pT1 sm1). However, even technically feasible, more invasive tumours (>pT1, sm2) are not considered for endoscopic resection, not because of technical aspects, but due to an uncalculated risk of LNM. A similar situation is given in advanced tumour stages (pT3 or higher) whenever a neoadjuvant treatment is recommended. With a more precise preoperative staging, in some of those patients an aggressive, potential toxic multimodal therapy could be prevented in case of exact knowledge of the lymph node status before treatment. Nowadays, imaging methods such as endoscopic ultrasound (EUS), computer tomography (CT) with/without combined positron-emission tomography (PET) or magnetic resonance imaging (MRI) are utilized for pretherapeutic evaluation. Nevertheless, all these procedures show a lack of accuracy in detecting local nodal metastases which consecutively leads to the dilemma how to clearly identify patients at risk. A recent study analysing 112 patients who either underwent primary surgery (n = 41) or neoadjuvant therapy (n = 71) due to OAC revealed a significant underestimation of the risk of LNM for EUS (p < 0.001), CT (p < 0.001) and PET/CT (p < 0.001). Accuracy, sensitivity and specificity of lymphatic metastases were 55.4%, 42.6% and 75% for EUS, 54.5%, 39.7% and 77.3% for CT and 57.1% 35.3%, and 90.9% for combined PET/CT^30^. Utilizing MRI technique for lymph node detection showed comparable accuracy for lymphatic involvement (66%)^31^. In contrast to this, Zhang *et al*. recently described high efficacy of EUS for detection of suspicious lymph nodes before surgical resection within their retrospective study on 112 OSCC patients^32^. The authors postulated an AUC value of 0.801 for EUS compared to the histopathological findings of the surgical specimens. However, EUS detection referred to morphological nodal aspects such as longitudinal diameter or echo pattern^32^ and therefore did not consider histological phenomena like extra-nodal metastases or nodal micro-metastases^7^. But not only evaluation of LNM is inaccurate by current methods, also detection of infiltration depth remains a problem especially among those patients under surveillance due to premalignant lesions such as Barrett oesophagus. A meta-analysis published by Qumseya *et al*. in 2018 including a total of 895 Barrett oesophagus patients from 11 studies demonstrated that the pooled false positive rate for estimating an advanced disease (pT1a/b) via EUS was 9.1% ([6.5–12.5%], p < 0.001) while the pooled false negative rate was 9.2% ([95%CI: 4.7–17.3%], p < 0.01)^33^. That means that almost twenty percent of Barrett oesophagus patients were wrongly evaluated and therefore received the non-optimal treatment significantly reducing the pooled accuracy of EUS to 74.6% ([58.7–85.8%], p = 0.004)^33^. Other studies supported these findings^34–36^. Additional (bio)markers are urgently needed. Therefore, we used miR-17 and miR-20a as independent predictors of LNM in this cohort of patients with OAC. The survival of the group with estimated LNM within the validation cohort was comparable to the actual follow-up of the group with histological proven LNM. The calculated parameters of our prediction model were as follows: AUC was 0.781 (95% confidence interval: 0.634–0.889) with p = 0.001. Specificity was 100% and sensitivity was 61.3%. Thus, our miR-based prediction model demonstrated comparable results to those clinical procedures during everyday routine.

In 2017, Liu *et al*. published a meta-analysis including 26 studies, which detected miR alterations in tissue, blood samples or both in a great variety of different human cancer types. They described that a high expression of the *miR-17-92* cluster predicted poor overall survival in Asian patients with OSCC. Within the six Caucasian cohorts no correlation was found^37^. A possible explanation for those results might be the focus on different subtypes of oesophageal cancer (OSCC versus OAC) and the relatively small percentage of Caucasian patients within the meta-analysis of Liu *et al*. In contrast to this, we demonstrated that upregulation of members of the *miR-17-92* cluster correlated with LNM in our OAC Caucasian study group with patients with pT2 OAC. The occurrence of LNM was associated with worse prognosis.

In 2014, Xu *et al*. found *miR-17*, *miR-18a* and *miR-19a* to be relevant predictive biomarkers in OSCC after analysing 105 surgical specimens and corresponding normal tissue utilizing qRT-PCR^38^. Concordant to our results, *miR-17* overexpression correlated with the occurrence of LNM (*p* = 0.035) and clinical stage (*p* = 0.022), while *miR-19a* upregulation was positively associated with tumour size (*p* = 0.005), clinical stage (*p* = 0.011), and LNM (*p* = 0.040)^38^.

Summarizing those studies, there is still a lack of information considering the *miR-17-92* cluster and its influence in OAC. In 2013, Wu *et al*. conducted a quantitative profiling of 754 human miRs in 35 normal epithelium, 34 Barrett’s oesophagus, and 36 OAC tissues, using the miR array approach^39^. They identified that an increase of *miR-106b-3p*, *miR-18*, *miR-18-3p*, *miR-20b* and *miR-92a-1-3p* were only found in OAC and was not detected in Barrett’s oesophagus tissues^39^. This leads to the conclusion, that the *miR-17-92* cluster might drive the OAC progression, supporting our results and further demonstrating upregulation within advanced tumour stages. However, there was no comparison to normal oesophageal mucosa within this current study.

To the best of our knowledge, this is the first study illustrating *miR-106a* to be associated with LNM and depth of tumour infiltration within OAC, although its role in tumorigenesis has been described within other malignancies before^29^. Interestingly, *miR-106a* has been shown to be upregulated in both gastric cancer FFPE tissue samples and gastric cancer cell lines^40^. *In vivo miR-106a* expression was positively associated with LNM (*p* = 0.002), vascular invasion (*p* = 0.017), and depth of infiltration (*p* = 0.009). Moreover, *miR-106a* promoted human gastric cancer cell migration and invasion *in vitro*. Furthermore, the tissue inhibitor of metalloproteinase-2 (*TIMP-2*) was identified to be the direct downstream target of *miR-106a*^40^. A knockdown of *TIMP-2* resulted in increased cell proliferation, migration, and invasion simulating the inhibitory effects of *miR-106a* on the expression of *TIMP-2*.

It is still uncertain which cellular pathways are affected by the dysregulation of the *miR-17-92* cluster within OAC. Some hints revealed that Proto-oncogene serine/threonine-protein kinase Pim-1 (*PIM-1)* is supposed to be involved in the transcriptional regulation of the *miR-17-92* cluster since silencing of this proto-oncogene caused decreased *miR17-92* expression in HeLa cells^41^. Additionally, overexpressing *miR-17-92* members resulted in the suppression of downstream targets such as tumour necrosis factor-α (*TNF-α*)^42^. Both *miR-17* and *miR-20a* have been shown to suppress cell migration and invasion of OSCC cells by modulating the *TGF-β/ITGB6* pathway^43^.

A few studies demonstrated the predictive value of circulating *miR-18a*, *miR-19a* and *miR-20a* within serum samples from OSCC patients^44–46^. Bai and co-workers analysed blood samples of healthy subjects in comparison to those patients with OSCC by qRT-PCR. Additionally, the authors compared the pre- versus postsurgical *miR-19a* levels showing high levels of *miR-19a* in cancer patients compared to normal probands and decreasing postsurgical *miR-19a* serum levels^44^. The findings of Hirajima *et al*. and He *et al*. confirmed the impact of the *miR-17-92* cluster in OSCC as non-invasive biomarkers^45,46^. Hirajima *et al*. initially examined *miR-18a* expression in human OSCC primary tissue, OSCC cell lines, and fibroblast cell lines by performing qRT-PCR assays. They described an increased *miR-18a* expression in both *in vivo* (*p* = 0.0020) and *in vitro* (*p* = 0.0121) OSCC samples. In the next step, serum samples of 106 OSCC patients were compared to 54 healthy volunteers, verifying these results. When performing an ROC curve analysis, considering *miR-18a* for detecting OSCC patients, the AUC was 0.9449^46^. For *miR-20a*, He and co-workers showed that plasma levels of *miR-20a* were significantly higher in OSCC patients (n = 70) than in healthy controls (n = 40), with an AUC of 0.767^45^. Contrary to the results of the current study, there was no significant correlation between *miR-20a* and the pathologic stage^45^. On the other hand as mentioned before, we did not include normal oesophageal tissue within our analysis and therefore cannot draw any conclusions considering the direct comparison between healthy mucosa and OAC. However, it should not be ignored that there is a limited comparability to the current study since both entities of oesophageal cancer (OAC and OSCC) seem to be biologically different.

Moreover, the *miR-17-92* cluster does not only seem to be feasible for evaluation of tumour progression and occurrence of LNM, but also could influence therapeutic decisions. Within an *in vitro* OAC/OSCC model of acquired chemotherapy resistance, miR profiles in cisplatin or 5-fluorouracil (5-FU) resistant variants versus chemotherapy sensitive control cells were compared using microarray and qRT-PCR^47^. The authors demonstrated that 5-FU resistant OAC as well as OSCC cells showed downregulated levels of *miR-18a-3p* (among other dysregulated miRs). Additionally, *miR-18a-3p* was decreased in 5-FU resistant OAC cells^47^. Interestingly, Hummel *et al*. reported that these findings were consistent with the expression of the putative target mRNA: While *miR-18a-3p* was decreased, its targeted mRNA KRAS was increased within 5-FU resistant OSCC cells^47^. Recently, Lynam-Lennon *et al*. analysed the resistance to neoadjuvant chemoradiation therapy in OAC by utilizing a radioresistant OAC cell line (subline of OE33) and endoscopic biopsies of patients prior to neoadjuvant treatment^23^. Their data suggested that *miR-17-5p* was decreased in radioresistant cells and that upregulation of *miR-17-5p* sensitized those radioresistant OAC cells to radiation. *In vivo miR-17-5p* was significantly decreased in pretherapeutic biopsies of those patients who showed poor response to neoadjuvant chemoradiation^23^.

Our analysis surely has its limitations being performed retrospectively and considering only a relatively small number of patients included within the examinations. Additionally, the study patients were recruited from three centres of upper gastrointestinal surgery resulting in a certain degree of heterogeneity. Another possible limitation lies in the lack of blood samples for evaluation of plasma levels of the identified miRs, especially according the chronological sequence including pre- and postsurgical samples. Nevertheless, all histopathological results were validated by an experienced gastrointestinal pathologist to guarantee a consistent assessment. Further, follow-up was worked out comprehensively over a relatively long period of time. In the future, we recommend further validation of our results by establishing a large prospective observational trial, recruiting a larger number of patients, and implementing both liquid biopsies, as well as FFPE tumour specimens. Besides patients who undergo primary surgery, patients who recieve different neoadjuvant therapies should also be considered. Additional *in vitro* experiments silencing those miRs or putative downstream targets might complement *ex vivo* examinations.

In conclusion, upregulation of *miR-17*, *miR-19a/b* and *miR-20a* as members of the *miR-17-92* cluster, as well as increased expression of their paralogue, *miR-106a*, were identified by comprehensive expression analysis to be associated with tumour progression and occurrence of LNM in human OAC, and therefore, might be feasible prognostic biomarkers for the future.

## Methods

### Patients

Surgical specimens analysed within the current study were derived from patients who underwent oesophagectomy between 2000 and 2010 in three centres for surgery of the upper gastrointestinal tract: (1) Department of General, Visceral and Cancer Surgery of the University Hospital of Cologne, (2) Department of General, Visceral and Transplant Surgery at the University Medical Centre of the Johannes Gutenberg-University Mainz, and (3) Department of Surgery, Horst Schmitt Kliniken Wiesbaden. The selection criteria for patients included were histologic proven OAC and primary surgery without neoadjuvant treatment. Patients with loco-regional LNM (pN+) and without (pN0) were included. The study was approved by the Institutional Ethics Committee of the University Hospital of Cologne and written informed consent was obtained from all patients. All experiments were performed in accordance with relevant guidelines and regulations.

### Detection, training and validation cohort

#### Detection group

The initial detection cohort was comprised of 12 patients with pT2 OAC who underwent primary oesophagectomy and from whom formalin-fixed paraffin-embedded (FFPE) tumour specimen samples were collected. This group of patients (pN0: n = 6 vs pN+: n = 6) was recruited for the miR array-based approach.

#### Training group

The miR data of the detection cohort was tested using qRT-PCR data of 43 patients forming the training cohort (including the 12 pT2 patients of the initial detection group). Additionally, this cohort was used for calculation of a prediction model.

#### Validation group

The miRs detected were used for validation using qRT-PCR in an independent group of 46 consecutive OAC patients with stage pT2 who underwent oesophagectomy at the Department of General, Visceral and Cancer Surgery of the University Hospital of Cologne. The prediction model established within the training cohort was also verified on the validation group.

### Staging and surgery

Routine staging diagnostics included esophagogastroduodenoscopy, endoscopic ultrasound, as well as a spiral contrast computer tomography of the thorax and abdomen.

The standard surgical procedure was laparotomic or minimally invasive gastrolysis and right transthoracic en bloc Ivor-Lewis oesophagectomy, including two-field lymphadenectomy of mediastinal and abdominal lymph nodes with gastric pull-up and intrathoracic anastomosis. Lymph nodes were removed from the resected oesophageal specimens for further histopathological examination according to a standardized protocol. The surgical procedure and lymph node preparation has been described previously^9^.

The patients’ outcome was followed up by regular visits within the local outpatient clinics of the participating centres.

### Histopathological procedure

Oesophageal adenocarcinoma specimens and resected lymph nodes were fixed within 5% formaldehyde and embedded in paraffin afterwards. Five µm thick slides were used for haematoxylin and eosin (HE) staining. If necessary, further staining with periodic acid/Schiff of the oesophagus specimens was performed to better evaluate the depth of tumour infiltration. All specimens were histopathologically analysed and classified by an experienced gastrointestinal pathologist according to the seventh edition of the Union for International Cancer Control/TNM-classification of malignant tumours including tumour localization, depth of tumour infiltration, grading, residual tumour as well as total number of resected and infiltrated lymph nodes^48^.

### Isolation of RNA from paraffin-embedded tissue

HE stained FFPE specimens were examined by an experienced pathologist (U.D. and A. Q.) for the presence of malignant tumour. Macrodissection was performed and a total of 60–80 µm of paraffin-embedded tissue per specimen was used for total RNA extraction. Paraffin extraction was performed by a 3-minute incubation in 320 µl of deparaffinization solution at 56 °C, followed by the addition of 240 µl of PKD buffer (Qiagen, Hilden, Germany) and 10 µl of proteinase K (Qiagen, Hilden, Germany). MiRs were isolated using miRNeasy FFPE Kit (Qiagen, Hilden, Germany) according to the manufacturer’s instructions. RNA quantity was estimated by A260-measurement using the ND-1000 NanoDrop spectrophotometer (NanoDrop, Wilmington, USA). 12 µl of total RNA (including miR) of 200–800 ng was isolated.

### MiR profiling of the detection cohort using MicroRNA Arrays

Comparative RT-PCR-based TaqMan low-density miR arrays A and B were applied for profiling of 3 μl samples containing 1000 ng of RNA. Reverse transcription reaction of the extracted RNA was performed using Megaplex RT Primer A and B and TaqMan™ microRNA RT Kit (Thermo Fisher Scientific, Darmstadt, Germany) as previously described^49^. 1 µg of cDNA was loaded on TaqMan™ Human MicroRNA Arrays Cards V2.0 (Set A and B) for miR profiling of 754 miRs with snoU6 as endogenous control (Thermo Fisher Scientific, Darmstadt, Germany). Quantification was performed by TaqMan™ ABI PRISM 7900HT Sequence Detection System (Thermo Fisher Scientific, Darmstadt, Germany). A total of 100 μl master mix containing 100 ng cDNA was loaded into each of the eight ports. The distribution into 48 reaction cavities per port was carried out by two short centrifugation steps (1 min 1200 rpm in a swinging bucket rotor, Heraeus-Multifuge-3S, Langensebold, Germany). Cross contamination was avoided by individual sealing of the 384 reaction cavities. Cycling conditions were as followed: Activation step at 92 °C for 10 min, followed by 40 PCR cycles at 95 °C for 1 sec and 60 °C for 20 sec.

### MiR quantification of the validation cohort by single real-time PCR

Candidate miRs identified by microRNA profiling were validated using qRT-PCR. 10 ng of whole RNA have been reverse-transcribed using cDNA by the TaqMan™ microRNA RT kit (Thermo Fisher Scientific, Darmstadt, Germany) in separate reactions made up of 3 µl of the specific primer *miR-17* (ID002308), *miR-19a* (ID000395), *miR-19b* (ID000396), *miR-20a* (ID000580), *miR-26a* (ID000405), *miR-106a* (ID002169) and *snoU6* snRNA (ID001973) as calibrator (all by Thermo Fisher Scientific, Darmstadt, Germany), 7 µl of the master mix (including 10X RT buffer, dNTP mix w/dTTp (100 M total), RNase inhibitor (20U/µL), MultiScribe™ RT enzyme (50U/µL) and nuclease-free water to scale the reaction up to a final volume of 15 µl. Parallel to this, 1 µg universal RNA from normal human tissues (# R4234565-1) (BioChain, Newark, USA) has been reverse-transcribed the same way. Reverse transcription was performed according to the instructions given by the manufacturer. Reaction conditions were 16 °C for 30 min, 42 °C for 30 min, 85 °C for 5 min and 4 °C until the end of the reaction.

Expression of *miR-17*, *miR-19a/b*, *miR-20a* and *miR-106a* was quantified using the QuantStudio 7 Flex Real-Time PCR System (Applied Biosystems, Darmstadt, Germany). The following reagents were used in single assays: *miR-17* (ID002308), *miR-19a* (ID000395), *miR-19b* (ID000396), *miR-20a* (ID000580), *miR-26a* (ID000405), *miR-106a* (ID002169), the *snoU6* snRNA (ID001973) in TaqMan Universal PCR Master Mix without AmpErase® UNG (Thermo Fisher Scientific, Darmstadt, Germany) and nuclease-free water for scaling up the volume to 20 µl. MiR levels were measured by their threshold cycle (Ct) and normalized by *snoU6* RNA as a calibrator. Therefore, cycle threshold (Ct) values were calculated into relative expression by standard curves for each specific primer in 1:2 serial dilutions of the reverse-transcribed universal RNA (# R4234565-1) (BioChain, Newark, USA). 1 µl containing 1 ng of sample cDNA was used in each 20 µl qRT-PCR assay. Real-time PCR quantification of miR-17, *miR-19a/b*, *miR-20a*, *miR-106a* and *miR-26a* was carried out in triplicates according to the manufacturer’s recommendations. Thermal cycling conditions were as followed: 50 °C for 2 min, 95 °C for 10 min, followed by 40 PCR cycles at 95 °C for 15 sec and 60 °C for 1 min.

### Statistical analysis

MicroRNA array data was quantified and normalized via comparative Ct-method using *snoU6* as an endogenous control. Relative miR expression was calculated as ΔCt (ΔCt = Ct_target miR_ − Ct_snoU6_) for each group (pN0 versus pN+) and fold changes were computed using the 2−ΔΔCt function. QIucore Omics Explorer software V 3.2 (QIucore, Lund, Sweden) was utilized to find variables that were best in separating pN+ versus pN0, presented as heatmap. Two-group comparison (two-sided T-test) was used, identifying data with a fold change of at least 1.5 and a p-value of <0.05.

Quantitative RT-PCR of the training and validation cohort was performed using single assays in triplicates, analysed via standard curve method. Relative quantification of each target miR (R) was calculated as the ratio of the target miR related to *snoU6* according to each cDNA sample.

Results of the miR expression profiles comparing pN+ vs. pN0 or pT-categories were graphically presented with Box-plots with a line representing the mean value. Correlation between nominal or ordinal parameters was tested using Chi-square test or Fisher’s Exact test. T-test or Mann–Whitney–test was utilized for comparison of two independent samples. Kaplan-Meier curves, with the log-rank test, were used for the analysis of survival prognosis. The level of statistical significance in all experiments was set to a *p*-value < 0.05.

Prediction of lymph node metastasis within the validation cohort was calculated by utilizing logistic regression analysis with backwards elimination of non-significant predictors for pN+ in those 43 patients with pT1, pT2 or pT3 OAC, including 18 cases with pN+ and 25 cases with pN0. Variables were either included if *p* < 0.05 or removed if *p* > 0.1. The statistical analysis was performed using the SPSS statistic program version 23 (SPSS, Chicago, USA). The graphical presentation of the designed predictive model and follow-up data was done with MedCalc Version 18.5 (MedCalc Software, Ostend, Belgium).

### Ethics approval and consent to participate

The authors state that they have obtained appropriate institutional review board approval for using the surgical specimens and have followed the principles outlined in the Declaration of Helsinki for all human or animal experimental investigations. All subjects provided written informed consent.

## Data Availability

The datasets generated and/or analysed during this current study are available from the corresponding author on reasonable request.
